# Alternative cDEP Design to Facilitate Cell Isolation for Identification by Raman Spectroscopy

**DOI:** 10.3390/s17020327

**Published:** 2017-02-09

**Authors:** Cynthia Hanson, Elizabeth Vargis

**Affiliations:** Department of Biological Engineering, Utah State University, Logan, UT 84322, USA; chanson8491@gmail.com

**Keywords:** contactless dielectrophoresis (cDEP), Raman spectroscopy, microfabrication

## Abstract

Dielectrophoresis (DEP) uses non-uniform electric fields to cause motion in particles due to the particles’ intrinsic properties. As such, DEP is a well-suited label-free means for cell sorting. Of the various methods of implementing DEP, contactless dielectrophoresis (cDEP) is advantageous as it avoids common problems associated with DEP, such as electrode fouling and electrolysis. Unfortunately, cDEP devices can be difficult to fabricate, replicate, and reuse. In addition, the operating parameters are limited by the dielectric breakdown of polydimethylsiloxane (PDMS). This study presents an alternative way to fabricate a cDEP device allowing for higher operating voltages, improved replication, and the opportunity for analysis using Raman spectroscopy. In this device, channels were formed in fused silica rather than PDMS. The device successfully trapped 3.3 μm polystyrene spheres for analysis by Raman spectroscopy. The successful implementation indicates the potential to use cDEP to isolate and identify biological samples on a single device.

## 1. Introduction

The use of label-free cell sorting, isolation, and identification techniques is becoming increasingly popular for analyzing biological samples. These techniques take advantage of cells’ intrinsic properties such as size, shape, or electrical polarizability to perform the required analyses. One label-free method of identification is Raman spectroscopy, which correlates inelastic light scattering with specific vibrational and rotational modes of the target molecule or cell. One common method for cell sorting and isolation prior to acquiring Raman spectra is dielectrophoresis (DEP), which is the phenomenon where a non-uniform electric field causes motion of a particle. When using DEP, the manipulation of particles is based on the applied electric field and the particles’ size, shape, and electrical properties.

There are several ways to implement DEP. A review of the mathematics of DEP and the various ways to implement DEP is beyond the scope of this article. However, several reviews and sources are available [1,2,3,4,5]. Briefly, in the early 1990s, DEP devices were made by embedding metallic electrodes within a sample chamber with a specific orientation or shape to create the non-uniform electric field. Unfortunately, these designs were prone to problems such as electrode fouling, electrolysis, Joule heating, and spatial limitations or how close the cells must be to the electrodes to be influenced by the electric field (approximately 30 μm). This limitation affects device efficiency and throughput. An alternative method to avoid common issues associated with DEP is insulator-based DEP (iDEP). In iDEP, electrodes are placed on opposite ends of a microfluidic device in direct contact with the sample solution. Insulating structures such as channel constrictions, sawtooth patterns, or an array of posts are placed within the channel between the electrodes. This arrangement forces the electric field to move around the structures, creating a non-uniform electric field required for DEP. Insulator-based DEP devices require high voltages to operate and are prone to electrolysis. Another DEP-based method that may address these drawbacks is contactless DEP (cDEP).

Contactless DEP creates a non-uniform electric field by insulating barriers within the sample channel as seen with iDEP. However, the electrodes in cDEP devices do not have physical contact with the sample channel. Instead, a thin insulating barrier separates liquid electrodes from the sample channel. This method is well-suited for biological samples as it minimizes the negative effects of electrolysis, electrode fouling, and Joule heating experienced by other common forms of DEP [3,6,7]. Fabricating a typical cDEP device involves polydimethylsiloxane (PDMS) casting on a silicon master mold (made previously using dry etching processes), removing the PDMS from the mold, and bonding the PDMS to glass [3]. The final structure requires a good seal of a thin PDMS membrane (~20 μm thick) to a glass substrate over 1–2 cm in length with typical channel depths of 50 μm. The device can be difficult to fabricate and replicate consistently as small defects during casting, de-molding, and bonding can occur, requiring many casts to produce one that will function properly.

Regardless of the way DEP is implemented, it is a powerful label-free tool to sort biological samples without tags, fluorescent markers, or specific DNA sequences for subsequent identification. It should be noted that DEP is not the only technique available for label-free means to sort and analyze cells. For example, laser tweezers Raman spectroscopy (LTRS) can trap, identify, and sort single cells [8,9,10]. Cells are targeted under a microscope and optically trapped using a laser. While trapped, a Raman spectrum is acquired to identify the cell. The cell can then be moved to another area based on the Raman spectra acquired. This technique is successful, but cannot meet the demands of high throughput as a Raman spectrum is acquired from each individual cell. In contrast, DEP can be used to automatically sort cells based on the cells’ intrinsic properties allowing for higher throughput.

To meet the need for isolation and identification, DEP is coupled with other techniques such as Raman spectroscopy (DEP–Raman spectroscopy) to isolate and identify biological samples. For example, several researchers have used a quadruple electrode arrangement to concentrate bacteria by negative DEP for Raman analysis [11,12,13]. Although successful, the design appears impractical as it is meant for small sample volumes (~200 μL) with some of the studies injecting even smaller volumes (10 μL) of concentrated bacteria at the DEP site for successful demonstration [11,12]. Not only is the sample size problematic, but the design is prone to common DEP-related issues as stated previously and is ill-suited to analyze samples containing more than one bacteria at a time. Other examples of DEP–Raman spectroscopy include sample labeling using Raman reporters or antibodies [14,15]. The use of labels increases costs, limits shelf life, and may result in wasted materials due to the broad range of bacteria strains that can be present in a sample. Label-free identification methods are appealing to cut costs, increase simplicity, and reduce the risk of false positives. In previous cases where DEP–Raman spectroscopy systems did not use labels or tags, the device was made using traditional metallic electrodes in contact with the sample channel [16,17], exposing the device to standard DEP problems of electrode fouling and electrolysis.

This article proposes a new design to improve operating parameters, address fabrication issues associated with cDEP, and allow for simultaneous acquisition of Raman spectra without interference from PDMS. Thus, the design offers label-free sorting and identification of a sample at the same time. The design was tested with polystyrene spheres as a proof of concept. Results indicate successful application of cDEP to trap particles for acquiring the Raman spectra. To the authors’ best knowledge, this is the first demonstration of using Raman spectroscopy and cDEP simultaneously.

## 2. Materials and Methods

### 2.1. Device Fabrication

**Figure 1:**
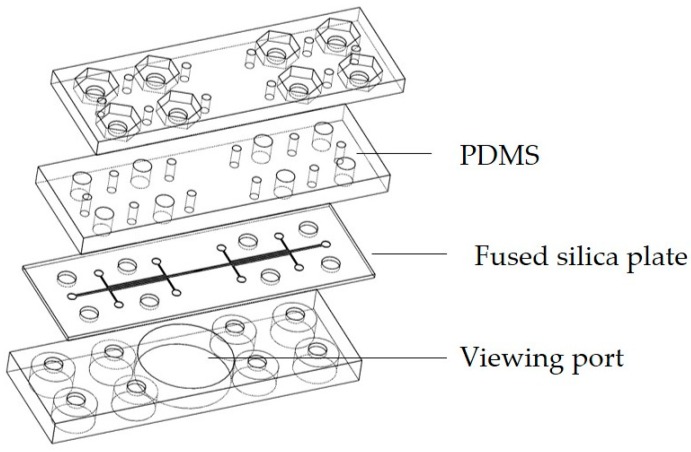
Illustration of the layered microfluidic device. Top and bottom plates were 3D printed with holes to accommodate #8-32 screws. The bottom plate is equipped with a viewing port for an inverted microscope. The second layer from the top is made of PDMS. The second plate from the bottom is the fused silica microfluidic plate.

The microfluidic device was constructed in a layered structure as illustrated in Figure 1. Outer plates were made using Stratasys VeraClear photopolymer and Objet260 Connex3 printer (Eden Prairie, MN, USA). The outer plates accommodate #8-32 screws to provide proper sealing of the device, as PDMS and fused silica do not bond easily.

The PDMS layer was made from Dow Corning 184 Sylgard (Auburn, MI, USA) silicone elastomer. A 10:1 ratio of PDMS monomer to curing agent was mixed, degassed, and poured onto a silicon wafer to provide a flat surface. The PDMS was cured at 100 °C for 35 min. After curing, the PDMS structure was carefully removed and trimmed. Holes were punched out using Miltex (Integra LifeSciences, York, PA, USA) 1.5 mm and 5 mm biopsy punches. The PDMS structure was aligned with the 3D printed plates and fastened to the fused silica microfluidic plate using #8-32 screws.

**Figure 2:**
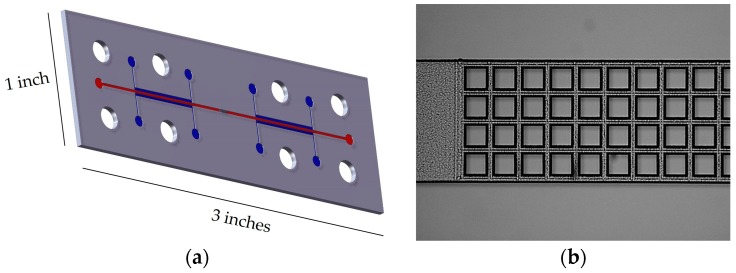
(**a**) Illustration of the fused silica microfluidic chip. Liquid electrodes and the sample channels are indicated by blue and red lines, respectively. The array of 4 by 15 square pillars act as insulating barriers in the middle of the sample channel. (**b**) Microscope image of the middle of the microfluidic sample channel showing the array of pillars in the middle of the sample channel. Pillar dimensions are 100 μm by 100 μm.

The fused silica microfluidic chip was fabricated by Translume (Ann Arbor, MI, USA). Figure 2a is an illustration of the microfluidic chip and Figure 2b is a microscope image of the sample channel with a square (100 μm by 100 μm) pillar array. The barriers between the liquid electrode and sample channels are 30 μm. The sample channel depth and width are 150 μm and 500 μm, respectively, with 20 μm set between each pillar in the array.

### 2.2. Sample Preparation

**Figure 3:**
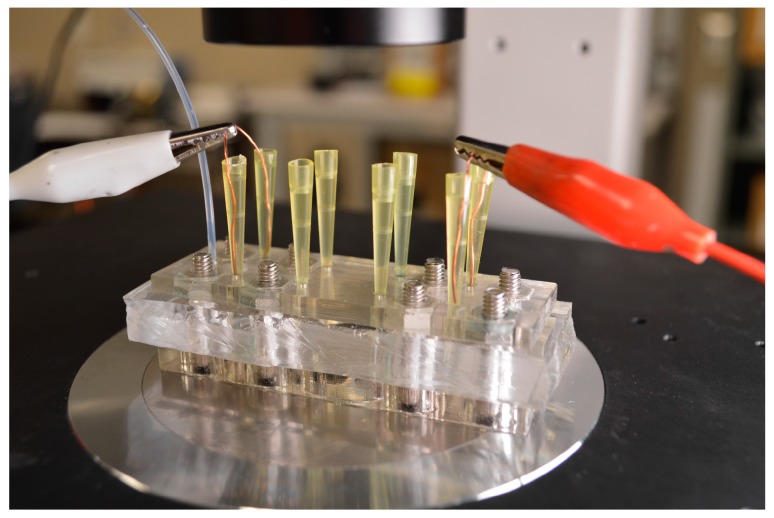
Image of the layered microfluidic device in operation set a-top an inverted microscope for analysis.

Polytetrafluoroethylene (PTFE—#20 AWG) tubing (Cole Parmer, Vernon Hills, IL, USA) was used to fill the sample and electrode channels. Two hundred microliter (Thermo Fisher Scientific, Waltham, MA, USA) pipette tips were trimmed to provide space for the microscope condenser and inserted through the PDMS to act as reservoirs for the liquid electrode channels. The sample consisted of 0.005× PBS (ScyTek, Logan, UT, USA), 0.1% TWEEN 20 (ScyTek), and 3.3 μm polystyrene fluorescent spheres (Thermo Fisher Scientific) at a concentration of approximately 2 × 10^7^ particles per milliliter. The sample had a conductivity of 40 μS/cm. Liquid electrodes were filled with 1× PBS with a conductivity of 15 mS/cm. Dilute 3,3′-diethylthiatricarbocyanine iodide (DTTC) dye (Sigma-Aldrich, St. Louis, MO, USA) at a 250 μM concentration in 1× PBS was added to the liquid electrodes to aid in visualizing during priming of the channels. The final concentration of DTTC in the channels was approximately 1 μM after inserting the pipettes filled with 1× PBS. Copper wires (28 gauge) were used to connect the electrodes spanning over the sample channel as shown in Figure 3.

### 2.3. Experimental Setup

A sinusoid wave was generated by an OWON AG1022 waveform generator (Industry, CA, USA). The signal was passed through a Trek Model 2205 high-voltage amplifier (Lockport, NY, USA) and monitored using an EZ Digital OS-5030 oscilloscope (Gyeonggi-do, Korea). The applied AC field (350 V_RMS_ at 100 Hz) was delivered to the device using alligator clips. The sample flow was controlled by a New Era Pump Systems, Inc. NE-300 syringe pump (Farmingdale, NY, USA) operating at 5 μL/h during analysis.

Raman spectra were collected using an in-house built Raman microscope unit as described and used previously [18,19]. The unit consists of an inverted Nikon Eclipse TE2000-S (Melville, NY, USA), a 785 nm single-mode laser (Innovative Photonic Solutions, Monmouth, NJ, USA), an IsoPlane 160 spectrometer equipped with a 1200 g/mm grating (Princeton Instruments, Trenton, NJ, USA), and a Pixis-400 CCD (Princeton Instruments). A 25 s integration time was used to acquire one spectrum. Spectra were processed using LightField (Princeton Instruments) and Renishaw Wire 4.1 (Gloucestershire, UK).

## 3. Results

### 3.1. Contactless Dielectrophoresis

**Figure 4:**
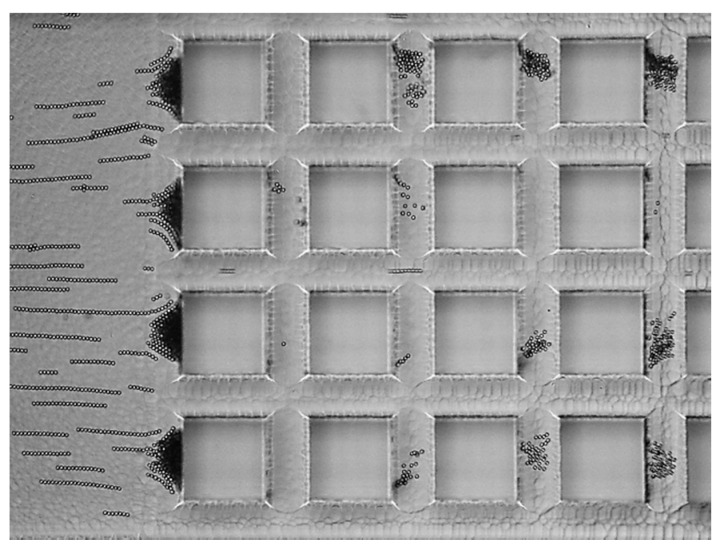
Image of cDEP device under operation (350 V_RMS_, 100 Hz, 5 μL/h), trapping polystyrene spheres with a diameter of 3.3 μm. Square pillars are 100 μm by 100 μm.

The cDEP device was used to demonstrate trapping of a sample containing fluorescent polystyrene spheres (~2 × 10^7^ particles/mL, PSS). Figure 4 is a 20× magnification of the device under operation. The applied AC field consisted of 350 V_RMS_ and 100 Hz, while the flow rate through the device was 5 μL/h. Particles were primarily trapped at the beginning of the pillar array. Trapping of the particles is necessary for subsequent evaluation using Raman spectroscopy. A video of the trapping process is included in the Supplementary Materials.

### 3.2. Raman Spectroscopy

**Figure 5:**
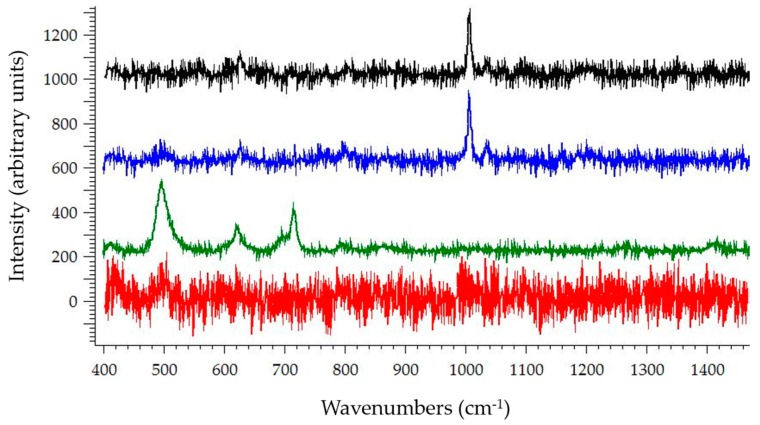
Raman spectra of 3.3 μm PSS trapped within the cDEP device (black), PSS on quartz coverslip (blue), PDMS (green), and the quartz coverslip with 0.005× PBS (red). The spectrum of PSS on a quartz coverslip (blue) is the positive control. The spectra of PDMS (green) and the quartz coverslip without PSS (red) are negative controls.

While the particles were trapped at the first set of pillars in the DEP device, a Raman spectrum was collected using a 785 nm wavelength laser at 15 mW for 25 s through a 40× objective lens. The resulting spectra were collected using LightField with a single 25-second acquisition and analyzed using Renishaw Wire 4.1. The spectrum of PSS trapped in the device was compared to positive and negative controls displayed in Figure 5 with a *y*-axis offset. From top to bottom, the spectra consist of PSS trapped under DEP, PSS on a quartz cover slip, PDMS, and the quartz coverslip with 0.005× PBS.

## 4. Discussion

This study successfully demonstrates a unique form of implementing cDEP, which provides several advantages over traditional cDEP fabrication methods. The microfluidic channels of a traditional cDEP device are in the PDMS structure itself, where the barriers between the liquid electrode and sample are composed of PDMS. In addition, traditional cDEP devices use PDMS structures or channel wall constrictions to form the non-uniform electric fields. This study used fused silica to form the barriers and insulating structures, while PDMS was used to seal the device. This design provides a greater voltage operating range and enhanced reusability.

Traditional cDEP devices are limited according to the dielectric breakdown of PDMS. Literature provides a wide range of dielectric breakdown values from 129 to 635 V/μm for thin membranes (2–20 μm) depending on PDMS thickness and electrode shape [20,21]. Yet, research articles concerning cDEP experiments report much lower dielectric breakdown values such as 20 V/μm [22] or 14 V/μm [23]. Table 1 lists publications that implemented cDEP for cell manipulations and includes the associated voltages, frequencies, and flow rates used. Most cDEP research articles do not use more than 250 V. To the authors’ knowledge, only one other research article has reported using 350 V during operation [24]. In [24], the device had a channel depth of 50 μm with microfluidic structures composed of PDMS at a 10:1 ratio. The barrier between the liquid electrodes and sample channel was made of PDMS with a 5:1 ratio 13 μm thick. The device was designed to prevent pearl chain formation, where particles are attracted to each other due to dipole–dipole interactions and are affected by particle size and concentration. They found that reducing pillars to sizes similar to target cells improved trapping efficiency and reduced pearl chaining. While the device from Čemažar et al. [24] has a high trapping efficiency and selectivity, it is only meant for isolation and enrichment before further off-chip analysis. The cDEP device presented here adds the advantage of on-chip isolation, enrichment, and analysis.

The applied voltage used in this paper was limited by the available equipment. With the aid of a step-up transformer or other equipment modifications as suggested in [25], higher voltages can be obtained without approaching the dielectric breakdown of fused silica (950 V/μm [26]) while maintaining a range of optimal and commonly used frequencies (1–1000 kHz [27]). Future work will make use of such equipment to demonstrate how fused silica can provide a higher range of applied voltage due to the dielectric breakdown. In addition to improved voltage range, the use of fused silica allows for acquisition of Raman spectra without interference of a PDMS signature as demonstrated in Figure 5. The device can be reused and therefore provide more reliable results.

It should be noted that the relationship between voltage and frequency requirements varies with cDEP device. Sano et al. [27] demonstrated that, for cDEP devices, voltage drop and associated electric field gradients can vary according to geometric configuration and applied frequency. Low frequencies cause a smaller percentage of the voltage drop to occur across the sample channel and therefore generate smaller DEP force vectors. In contrast, the use of higher frequencies causes a higher percentage of the voltage drop to occur within the sample channel, therefore lowering the voltage demand. Our study used a very low frequency (100 Hz) to demonstrate separation of particles due to negative DEP (particles drawn to areas with a low electric field gradient). Although the frequency used in this study was considerably lower than what is used for typical separations, it demonstrated that DEP separation can be achieved even under unfavorable conditions.

Future work will address issues raised from the current design. For example, the flow rate used for this study was the same or slower than other research articles, as listed in Table 1. To be competitive at providing rapid analyses, a faster flow rate will need to be achieved. Device features such as pillar size, shape, and spacing will also be changed to accommodate 1-μm-sized particles, as the end goal is to trap bacteria and prevent pearl chain formation. The current setup primarily traps particles at the first column of pillars with secondary trapping within the array likely due to pearl chain formations. As the design was created as a proof of concept to perform cDEP and Raman spectroscopy simultaneously, the authors acknowledge that the arrangement has not been optimized for trapping efficiency. To improve trapping efficiency and selectivity, smaller pillar sizes will be incorporated with columns of pillars spaced further away from each other in future devices. In addition, structures will need to be arranged for simultaneous separation of multiple particles in a sample as demonstrated in other studies [35,36].

## 5. Conclusions

A cDEP device was successfully fabricated which demonstrated simultaneous trapping and Raman analysis of 3.3 μm polystyrene spheres. The device is constructed with microfluidic channels etched into fused silica, allowing for a greater voltage operating range and improved reusability compared to typical cDEP designs. To the authors’ knowledge, this article presents the first demonstration where Raman spectroscopy was performed on a cDEP device.

## Figures and Tables

**Table 1:** List of cDEP publications and their associated operating parameters. As the list consists of alternating current sources, voltage is expressed in root mean square (V_RMS_).

Source	Voltage (V_RMS_)	Frequency (kHz)	Flow Rate (μL/h)
[28]	200	5–50	5
[29]	200	5–70	5
[30]	250	500	Not reported ^1^
[22]	250	600	1000
[27]	227–250	50–100	10
[31]	20–50	120–320	20
[23]	70–190	300	20
[32]	20–150	140–500	20
[6]	250	85	10–15
[33]	200–300	10–70	5
[34]	50–200	200–600	20
[24]	250–350	30	1200–2160
Current article	350	0.1	5

^1^ Rate driven by electrokinetic flow.

## Supplementary Materials

The following video is available online at http://www.mdpi.com/1424-8220/17/2/327/s1, Video S1: Trapping of polystyrene microspheres using contactless dielectrophoresis.
